# Perturbed cholesterol and vesicular trafficking associated with dengue blocking in *Wolbachia*-infected *Aedes aegypti* cells

**DOI:** 10.1038/s41467-017-00610-8

**Published:** 2017-09-13

**Authors:** Vincent Geoghegan, Kirsty Stainton, Stephanie M. Rainey, Thomas H. Ant, Adam A. Dowle, Tony Larson, Svenja Hester, Philip D. Charles, Benjamin Thomas, Steven P. Sinkins

**Affiliations:** 1 MRC-University of Glasgow Centre for Virus Research, University of Glasgow, Glasgow, G61 1QH UK; 2 Biomedical and Life Sciences, University of Lancaster, Lancaster, LA1 4YQ UK; 3 Bioscience Technology Facility, Department of Biology, University of York, York, YO10 5DD UK; 4 Sir William Dunn School of Pathology, University of Oxford, Oxford, OX1 3RE UK; 5 Present Address: Fera Science Ltd, Sand Hutton, York YO41 1LZ UK

## Abstract

*Wolbachia* are intracellular maternally inherited bacteria that can spread through insect populations and block virus transmission by mosquitoes, providing an important approach to dengue control. To better understand the mechanisms of virus inhibition, we here perform proteomic quantification of the effects of *Wolbachia* in *Aedes aegypti* mosquito cells and midgut. Perturbations are observed in vesicular trafficking, lipid metabolism and in the endoplasmic reticulum that could impact viral entry and replication. *Wolbachia-*infected cells display a differential cholesterol profile, including elevated levels of esterified cholesterol, that is consistent with perturbed intracellular cholesterol trafficking. Cyclodextrins have been shown to reverse lipid accumulation defects in cells with disrupted cholesterol homeostasis. Treatment of *Wolbachia-*infected *Ae*. *aegypti* cells with 2-hydroxypropyl-β-cyclodextrin restores dengue replication in *Wolbachia*-carrying cells, suggesting dengue is inhibited in *Wolbachia-*infected cells by localised cholesterol accumulation. These results demonstrate parallels between the cellular *Wolbachia* viral inhibition phenotype and lipid storage genetic disorders.

## Abstract (web summary)

*Wolbachia* infection of mosquitoes can block dengue virus infection and is tested in field trials, but the mechanism of action is unclear. Using proteomics, Geoghegan et al. here identify effects of *Wolbachia* on cholesterol homeostasis and dengue virus replication in *Aedes aegypti*.

## Introduction

W*olbachia* are intracellular endosymbiotic bacteria of invertebrates that are maternally transmitted. They can manipulate host reproduction to allow rapid population spread, especially using cytoplasmic incompatibility, a crossing sterility that can provide a reproductive advantage to females carrying the bacteria^1^. In certain host-strain combinations where high bacterial densities are reached, *Wolbachia* can significantly reduce the transmission of some of the most important mosquito-borne pathogens of humans including dengue virus (DENV) and chikungunya virus^2–13^. The *w*Mel strain has been taken to very high population frequency in wild populations of *Aedes aegypti* in field trials^14^, and is now being deployed on a larger scale in a number of countries for dengue control. It is important to gain a full understanding of the mechanisms by which *Wolbachia* inhibit arbovirus transmission, in order to be able to maximise the effectiveness and longevity of its use in dengue control—in particular, to be able to rapidly understand, and react effectively to, any operational failures that may arise over time.

Possible contributory factors to the phenotype include immune activation and elevated reactive oxygen species as observed in *Ae*. *aegypti* ^4, 15^, but virus inhibition can apparently occur without observable immune activation in *Ae*. *albopictus* ^16^ and *Drosophila* ^17^. *Wolbachia* likely incorporate cholesterol into their membranes as a substitute for lipopolysaccharide^18, 19^, and it has been hypothesised based on *Drosophila* data that direct competition between the bacterium and viruses for this resource may be responsible for the dengue transmission-blocking phenotype^20^, although no supporting evidence in mosquitoes has to date been presented.

To characterise the effects of a high-density virus-blocking *Wolbachia* infection in *Ae*. *aegypti* and detect changes in host cellular pathways that may play a role in limiting viral replication, we performed a quantitative proteomics analysis of *w*MelPop versus uninfected Aag2 cells. We detected perturbations in vesicular trafficking, the endoplasmic reticulum (ER) and lipid metabolism associated with *Wolbachia* infection. Differential expression of key proteins involved in cholesterol homeostasis indicated that *Wolbachia-*infected cells experience localised cholesterol accumulation and a defect in intracellular cholesterol transport. Treating *Wolbachia-*infected cells with 2-hydroxypropyl-β**-**cyclodextrin, used to restore cholesterol homeostasis in Niemann–Pick type C cells, rescued dengue virus replication. This suggested some similarities between *Wolbachia* infection and human lipid storage disorders such as Niemann–Pick type C.

## Results

### Proteomic analysis of *Wolbachia-*infected cells

To characterise the effect of *Wolbachia* infection on the host, we carried out a quantitative proteomic analysis of uninfected and *w*MelPop-infected *Ae*. *aegypti* cells. In the *w*MelPop-carrying cell line, differential expression of 265 host proteins was observed compared to *Wolbachia*-free cells (Table 1, Supplementary Fig. 1, Supplementary Tables 1 and 2 and Supplementary Datas 1, 2). Perturbations in vesicular trafficking, the ER as well as alterations in lipid/cholesterol metabolism were particularly notable in *Wolbachia-*infected cells (Table 1). Differential expression of several host proteins involved in lipid metabolism was detected including upregulation of fatty acid desaturase, which would compensate for the uptake of unsaturated fatty acids by *Wolbachia*, and proteins involved in catabolisis of fatty acids (saposin, phytanoyl-CoA hydroxylase). Conversely, fatty acid synthase was downregulated; in the presence of DENV, fatty acid synthase is upregulated and required for efficient replication^21^, and hence this change is likely to be antagonistic to DENV replication (Supplementary Table 2).

**Table 1:** Presence of *Wolbachia* causes differential expression of host proteins with roles in the unfolded protein response, vesicular trafficking, lipid metabolism and autophagy

	

Proteins in an *Ae*. *aegypti* cell line infected with *w*MelPop were quantified relative to uninfected cells. Fold changes in *w*MelPop relative to uninfected cells are coloured according to increased (blue) or decreased (red) levels

**Fig. 1:**
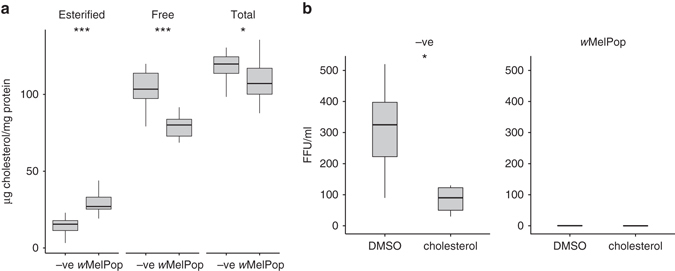
Infection with *Wolbachia* is associated with elevated esterified cholesterol but lower free cholesterol. **a** Quantification of esterified, free and total cholesterol in uninfected (–ve) and *w*MelPop-infected Aag2 cells. Quantification was performed on 12 biological replicates of each cell line. **b** DENV replication in –ve or *w*MelPop-infected Aag2 cells. Cells were pretreated with DMSO or 50 µg/ml cholesterol for 48 h prior to infection with DENV at a multiplicity of infection of 0.1. Cell culture supernatant was harvested 5 days post infection and DENV viral titre (fluorescent focus units/ml) measured by fluorescent focus assay. Statistical significance was determined with Student’s *t*-test, *n* = 5

A lysosomal protein (ncu-g1) and syntaxin-17 (involved in autophagosome–lysosome fusion) and regulators of endocytosis (wurst and rush hour) were all upregulated in the presence of *Wolbachia*, while two proteins involved in exocytosis and ER to Golgi transport (rabkinesin-6 and tomosyn) were downregulated (Supplementary Table 2). Further evidence for perturbations in vesicular trafficking were observed as alterations in the levels of mucolipin-3, vacuolar sorting protein, syntenin, ndrg1 and myosin V (Fig. 1). Decreased levels of the V-ATPase regulatory subunit c in *Wolbachia*-infected cells could affect clathrin-mediated endocytosis and endosomal acidification, which are critical for DENV entry^22^. Autophagy, the homeostatic process of encapsulation of cytoplasmic components in double-membrane vesicles for degradation, appears to be suppressed in *Wolbachia-*infected cells since ATG4B and TSC2 are downregulated. Autophagy is activated and required by DENV for triglyceride release from storage in cytoplasmic lipid droplets to provide energy for replication via β-oxidation^21^, a subversion of its usual anti-pathogen role. In contrast, *Wolbachia* density can be reduced by autophagy activation^23^, providing a selective pressure on the bacterium to suppress this pathway, with a probable antiviral side effect. Two lysosomal proteins, ncu-g1 and saposin, were upregulated, which may be due to the inhibition of autophagy causing an accumulation of lysosomes.

ER proteins with roles in protein folding and glycosylation were upregulated in *Wolbachia-*infected cells (Table 1), suggesting the presence of ER stress and triggering of the unfolded protein response, a pathway aimed at increasing protein folding capacity to restore ER homeostasis^24^. Members of the uridine diphosphate glycosyltransferase family were upregulated, which play an important role in quality control of glycoproteins in the ER^25^. Components of the oligosaccharyltransferase complex were upregulated in *Wolbachia-*infected cells, involved in the co-translational glycosylation of proteins as they enter the ER^26^. A major ER calcium-dependent chaperone, calnexin 99a, was upregulated as well as a protein disulfide isomerase, indicating a cellular attempt to increase protein folding activity.

Several proteins that are known to respond to changes in cholesterol or play a role in cholesterol metabolism were differentially regulated. Apolipoprotein D and ABCA1 (cholesterol efflux) and a homologue of cholesterol transporter NPC2 were upregulated in the presence of *Wolbachia*, while the low-density lipoprotein (LDL) receptor (cholesterol import) was downregulated (Supplementary Table 2). When cells sense an excess of cholesterol, the LDL receptor and fatty acid synthase are downregulated and cholesterol efflux transporters are upregulated. In human Niemann–Pick disease, cholesterol and/or sphingolipids accumulate in late endosomes/lysosomes due to mutations in the intracellular cholesterol transport proteins NPC1 or NPC2^27^, and is accompanied by an increase in Apolipoprotein D expression. These cellular responses to cholesterol accumulation/overload mirror the protein expression changes observed in *Wolbachia-*infected cells, consistent with the host cell experiencing a defect in intracellular cholesterol transport.

### Perturbed cholesterol homeostasis in *Wolbachia-*infected cells

Based on the proteomic results, cholesterol levels were measured and *Wolbachia-*infected cells were found to contain ~100% more esterified cholesterol compared to uninfected cells (Fig. 1a, *p* = 0.000022, Student’s *t-*test), although free cholesterol levels were decreased by ~24% in *Wolbachia-*infected cells (*p* = 0.000047, Student’s *t-*test). Total cholesterol levels were ~9% lower in *Wolbachia-*infected cells (*p* = 0.032, Student’s *t-*test). The increase in levels of esterified cholesterol in infected cells is consistent with hypercholesterolaemia, since cholesteryl esters are the primary storage and transport forms of cholesterol^28^. Supplementation of Aag2 *w*MelPop cells with cholesterol did not rescue DENV replication (Fig. 1b), while in uninfected Aag2 cells cholesterol supplementation inhibited DENV replication, as previously reported^29^.

Perturbed cholesterol homeostasis can cause ER stress^30^, but the reverse is also true, with ER stress being associated with lipid dysregulation and an increase in cellular lipid droplet content^31^. It is therefore difficult to precisely dissect the hierarchy of perturbations in *Wolbachia-*infected cells; however, it is likely that both ER stress and disrupted cholesterol homeostasis negatively impact DENV replication.

There is evidence that cholesterol plays an important role in entry of DENV and other flaviviruses to mammalian cells and is required in the DENV viral envelope^29, 32^; it is also likely that they require particular lipid and cholesterol profiles for exit from the cell^33^. Specific cellular cholesterol profiles are required by arboviruses and both cholesterol depletion and excess can inhibit DENV—the latter probably by changing membrane rigidity^29^. The 2-hydroxypropyl-β-cyclodextrin (2HPCD) is a cholesterol-binding agent routinely used to modulate cellular cholesterol content^34^, depending on the cyclodextrin/cholesterol molar ratio, ranging from cholesterol depletion to enrichment. At high concentrations (>1 mM), it acts as a cholesterol sink and can extract cholesterol from membranes, while at lower concentrations, it can also act as a cholesterol shuttle, transporting cholesterol between membranes^35–37^. At lower concentrations (<1 mM), 2HPCD shows efficacy in the treatment of mouse models of Niemann–Pick type C disease^38^, probably by releasing trapped cholesterol into the cytosol^33^.

### DENV rescue in *Wolbachia-*infected cells with 2HPCD

**Fig. 2:**
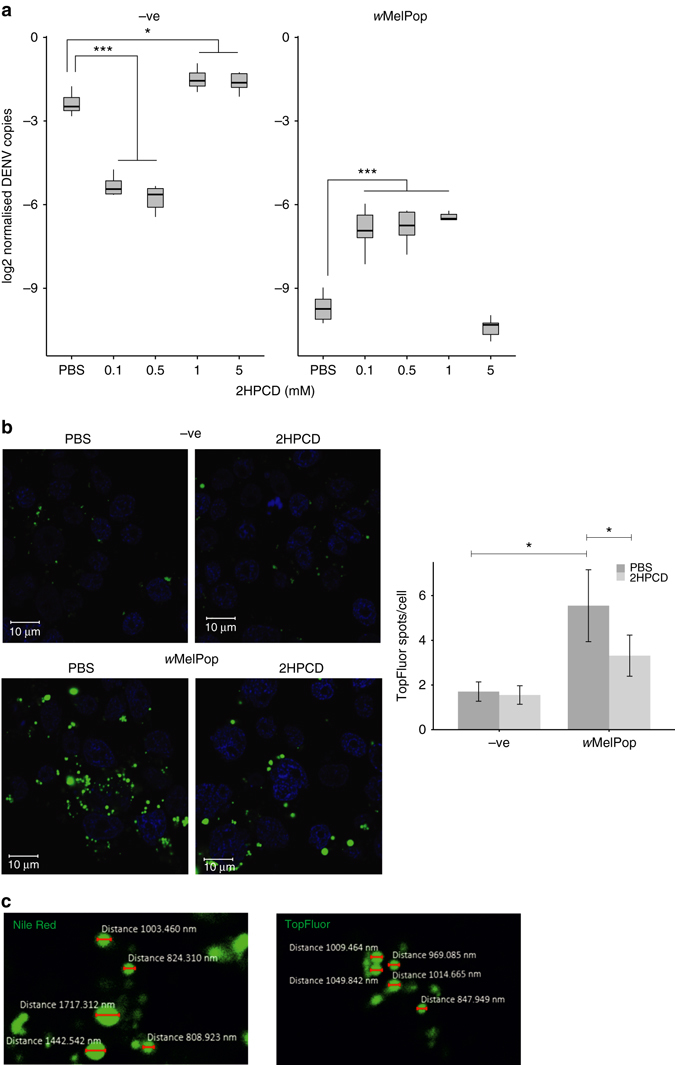
Pretreatment of *Wolbachia-*infected Aag2 cells with low concentrations of 2-hydroxypropyl-β-cyclodextrin (2HPCD) reverses inhibition of DENV replication. **a** Uninfected cells (–ve) and *w*MelPop-infected cells were treated for 48 h with various concentrations of 2HPCD prior to addition of DENV virus at a multiplicity of infection of 0.1. Cells were harvested 5 days post infection and DENV levels measured by qPCR. Significant differences of <0.05 are indicated as determined by analysis of variance (ANOVA) corrected by Tukey’s HSD test, *n* = 5. **b** Cholesterol turnover imaged using TopFluor cholesterol. Uninfected and *w*MelPop-infected cells were pretreated with either PBS or 0.1 mM 2HPCD for 24 h. Cells were then labelled with TopFluor cholesterol for 30 min and subsequently incubated with either PBS or 0.1 mM 2HPCD for 24 h before imaging by fluorescence confocal microscopy; *scale bar* indicates 10 µm. Lipid droplets were quantified as TopFluor-positive spots/cell; *error bars* denote s.d. Statistical significance was assessed by ANOVA corrected by Tukey’s HSD test, *n* = 5. **c** Imaging of Nile Red stained or TopFluor-treated *w*MelPop cells and measurement of lipid droplet size

Our results showed upregulation of a Niemann–Pick type C-related protein and an accumulation of stored cholesterol in *Wolbachia-*infected cells. 2HPCD was therefore added to *Ae*. *aegypti* cells, and rescue of DENV replication in *w*MelPop-containing cells was observed (Fig. 2a). In contrast, the same concentrations of 2HPCD strongly inhibited DENV in control *Wolbachia*-free *Ae*. *aegypti* cells (Fig. 2a), in agreement with a previous study^32^. Importantly, *Wolbachia* density was not significantly affected by the cyclodextrin treatment, thus ruling out a density-mediated mechanism of rescue of inhibition (Supplementary Fig. 2). 2HPCD also rescued DENV replication in *w*MelPop-containing cells at the infectious particle level (Supplementary Fig. 3). To visualise cholesterol dynamics, pulse labelling of uninfected and *w*MelPop-infected Aag2 cells with a fluorescently labelled cholesterol analogue was undertaken and the cells imaged 48 h later. The *w*MelPop cells showed significantly higher intracellular accumulation of the fluorescent cholesterol in large spots within the cell (Fig. 2b), and this accumulation was reduced upon 2HPCD treatment. The labelled cholesterol did not colocalise with the rab7 marker of late endosomes (Supplementary Fig. 4). We hypothesised that TopFluor was localising to lipid droplets due to the round shape and size of the spheres observed. In order to determine if this was the case, we stained *w*MelPop cells with the dye Nile Red. This dye specifically binds lipids and fluoresces green/yellow when binding neutral lipids such as those stored in lipid droplets^39^. Comparison of the spheres observed under Nile Red and TopFluor staining (Fig. 2c) indicate that TopFluor is localising to lipid droplets. Further to this, three-dimensional (3D) reconstruction of TopFluor labelled images showed, upon 2HPCD treatment, a clear diffusion of green fluorescence observed in *w*MelPop cells and not in negative cells, suggesting a reduction in lipid droplet accumulation of TopFluor (Supplementary Fig. 5).

### Proteomic analysis of *Wolbachia-*infected *Ae*. *aegypti* midguts

In order to examine whether similar *Wolbachia*-induced changes could be observed in vivo, a quantitative proteomics analysis of uninfected and *w*Mel-infected *Ae. aegypti* midguts was performed. The *w*Mel strain is a less efficient dengue blocker than *w*MelPop and is maintained at a lower density, but is currently the strain most commonly being utilised for field releases^5, 9–13^. As midguts are the first tissue encountered by DENV following its ingestion in a bloodmeal, any *Wolbachia-*associated perturbations would significantly impact on DENV replication and dissemination in the host. In total, 3,562 unique proteins were quantified, of which 434 showed differential expression in *w*Mel vs. uninfected midguts (Table 2, Supplementary Table 3 and, Supplementary Data 3). *Wolbachia-*associated changes were observed in cholesterol homeostasis, ER proteins, vesicular trafficking and autophagy, suggesting similar perturbations were induced by *Wolbachia* in the midguts and cell line (Table 2).

**Table 2:** Differentially expressed proteins with roles in the unfolded protein response, vesicular trafficking, lipid metabolism and autophagy in *w*Mel-infected vs. wt *Aedes aegypti* midguts

	

Fold changes in *w*MelPop relative to uninfected cells are coloured according to increased (blue) or decreased (red) levels

In *w*Mel-carrying midguts there was upregulation of several Niemann–Pick proteins and proteins involved in intracellular sterol transport, with the exception of sterol carrier protein X, which was downregulated. As observed in the cell line, several ER proteins were upregulated including components of the oligosaccharyl transferase complex (stt3b, DAD1) and proteins responsible for restoring ER homeostasis as part of the unfolded protein response (RtcB, calnexin 99a, heat shock protein, peptidylprolylisomerase). Interestingly, several secreted proteins (trypsin 5G1, chymotrypsin-1, chymotrypsin-2, vitellogenin, Supplementary Data 3) were upregulated in the *Wolbachia-*carrying midguts. These proteins are normally upregulated following a bloodmeal; their upregulation in non-blood-fed *Wolbachia-*infected mosquitoes could be a result of a defect in protein secretion, possibly as a result of altered ER homeostasis, leading to intracellular accumulation. Elevated trypsin 5G1 prior to a bloodmeal may further reduce DENV entry and replication in the midgut^40^.

Several proteins whose human homologues are mutated in lysosomal storage disorders were found to be upregulated in the presence of *Wolbachia*, providing further evidence of perturbed lipid metabolism/vesicular trafficking (Supplementary Table 3). In particular, sphingomyelin phosphodiesterases, enzymes deficient in Niemann–Pick disease type A^41^, were upregulated. These data strongly suggest that the perturbations observed in the cell line also occur *in vivo*, and also demonstrate that they are not restricted to the *w*MelPop infection, which reaches unusually high density and affects the transcription of an unusually large number of host genes compared to lower-density virus-blocking *Wolbachia* infections^42^.

## Discussion

A number of the proteins affected by the presence of *Wolbachia* are mutated or deficient in human lysosomal storage disorders such as Niemann–Pick disease, suggesting some parallels between these cellular states. As is thought to be the case in 2HPCD treatment of the Niemann–Pick type C disorder, the cyclodextrin treatment of *Wolbachia-*infected cells is likely to act as a shuttle and release trapped cholesterol into the cytosol, facilitating virus replication. Our data suggest *Wolbachia-*mediated inhibition occurs early in the DENV replication cycle, consistent with a recent study by Rainey et al.^43^.

Since lipids are degraded in late endosomes, perturbations in vesicular trafficking at this stage would likely manifest as a dysregulation of several lipid classes^44^, and manipulation of other lipids should be further investigated in future work. Molloy et al.^45^ performed lipidomic analysis of *Wolbachia-*infected *Ae*. *albopictus* cells revealing depletion of ceramides and altered levels of some sphingomyelin species. Data presented here suggest this may be related to defects in late endosome processing of sphingomyelin to ceramide. *Wolbachia-*infected cells showed an upregulation of sphingomyelin phosphodiesterases, the enzyme responsible for hydrolysing sphingomyelin to ceramide, indicating a host attempt to correct defects in late endosome lipid processing and restore cellular homeostasis. Molloy et al.^45^ also found that phosphatidylcholine and phosphatidylethanolamine levels were strongly perturbed. Treatment of cells with U18666A induces a Niemann–Pick type C-like phenotype, inhibits Dengue replication and similarly causes dysregulation in phosphatidylcholine and phosphatidylethanolamine due to defects in late endosome lipid trafficking^46^.

In contrast to the *Aedes* data presented here, supplementation with cholesterol reduced *Wolbachia*-mediated protection from the pathogenic virus DCV in *Drosophila* and increased viral replication^20^. Given that this virus is not related to the Flavivirus DENV, there are likely to be differences in the mechanisms of cell entry/replication and degree of cholesterol dependence that will influence the interactions between virus, *Wolbachia* and host cells. In addition, the effect of cholesterol supplementation on DCV titre in uninfected *Drosophila* was not investigated in the study of Caragata et al.^20^, making it unclear if the effect observed was a reversal of *Wolbachia-*mediated inhibition or a general enhancement of viral replication. The differences observed between *Drosophila* and *Aedes* could also be related to fluctuations in *Wolbachia* density following dietary cholesterol treatment in *Drosophila*, rather than a direct effect^20^.

In both the *Wolbachia-*infected *Ae*. *aegypti* cell line and midguts, perturbations were observed in the ER, vesicular trafficking, lipid metabolism and autophagy. The good overlap between the two data sets at the level of pathways affected but not at the level of individual differentially expressed proteins is likely due to the different proteomic composition of a cell line vs. midguts and the different lysis and quantitation methods used.

The detected ER perturbation in *Wolbachia-*infected cells is of interest given that DENV uses the ER for synthesis and maturation of proteins^47^ and data show close association of *Wolbachia* with the ER membranes^48^. Lipid droplets are ER-derived protein and lipid storage organelles targeted by DENV structural protein C^49^; therefore, the dysregulated cholesterol trafficking and accumulation of cholesterol rich lipid droplets observed in *Wolbachia-*carrying cells may interfere with proper localisation of DENV protein C, impairing replication. Cholesterol-rich lipid droplets were dispersed after 2HPCD treatment and 2HPCD has been widely used to modulate cholesterol homeostasis; however, it should be noted that 2HPCD contains a hydrophobic core which may bind to lipids other than cholesterol.

Overall, the data presented demonstrate that in *Ae*. *aegypti* cells, *Wolbachia* infection interferes with intracellular cholesterol trafficking, leads to an accumulation of intracellular cholesterol stores, perturbs the ER and interferes with vesicular trafficking and lipid metabolism. Treatment with cyclodextrin provides a clear indication that these changes are antagonistic to DENV.

## Methods

### Cell culture

*Ae*. *aegypti* Aag2 cells (origin J. Peleg, Israel Institute for Biological Research, Israel, 1970s; obtained from A. Kohl, Glasgow University) were cultured at 28 °C in Schneider’s *Drosophila* medium (Pan Biotech Ltd) supplemented with 10% fetal bovine serum (FBS; Sigma) and 200 units/ml penicillin 200 μg/ml streptomycin (Sigma). An Aag2 subline was transinfected with *w*MelPop *Wolbachia* from *Drosophila melanogaster* line *w* ^*1118*^, as previously described^16, 50^. Briefly, *w*MelPop-infected *D. melanogaster* embryos were collected and dechorionated using a 2.1% sodium hypochlorite solution for 2 min. Embryos were rinsed three times with distilled water, immersed in 70% ethanol for 15 s and washed three times with phosphate-buffered saline (PBS). Embryos were homogenised in a mini Dounce homogeniser (Wheaton) using a tight pestle for 2–3 min. Embryo homogenate was overlaid on 75% confluent Aag2 cells in 12-well plates. Plates were centrifuged at 2,000×*g* for 1 h at room temperature (RT) and cells subsequently incubated for 24 h at 28 °C. Absolute *Wolbachia* density in the Aag2 *w*MelPop line was 106 ± 30 (s.d., *n* = 3) Wolbachia surface protein/homothorax (*wsp*/*HTH*) as measured by quantitative PCR (qPCR; see qPCR methods). Vero cells (ECACC catalogue no. 85020205) were cultured at 37 °C with 5% CO_2_ in Dulbecco’s modified Eagle’s medium (DMEM) (Sigma) with 2 mM l-glutamine and 10% FBS in a humidified incubator.

### Experimental design and statistical rational

A total of four independent biological replicates (flasks) were processed, sufficient to estimate differential fold changes using the LIMMA software package^51^. Label swapping was performed for each biological replicate to eliminate systematic label quantification bias. Five independent biological replicates of uninfected and *w*Mel-infected *Ae*. *aegypti* midguts were tandem mass tag (TMT) labelled (Thermo) and processed for mass spectrometry, sufficient to estimate statistically significant fold changes using ScaffoldQ+ 4.0 (Proteome Software Inc.).

### Cell lysis, digestion and peptide labelling

Cells were harvested by scraping at 50% confluency (15 × 10^6^ per replicate, 4 replicates per cell line), washed twice in PBS and stored at −80 °C. Cell pellets were thawed on ice and lysed in 300 µl 0.2% PPS silent surfactant (Expedeon) in 50 mM triethylammonium bicarbonate (TEAB) and vortexed. Lysates were heated at 95 °C for 5 min, cooled on ice and sonicated with a microtip sonicator 3 times for 10 s each. Protein was measured using the bicinchoninic acid (BCA) assay (Pierce). Samples were reduced with 5 mM dithiothreitol (DTT) for 30 min at 50 °C and alkylated with 15 mM 2-Chloracetamide for 30 min in the dark. Proteins were digested overnight at 37 °C with trypsin (Sigma)/protein of 1:75 in the presence of 1 mM CaCl_2_. PPS surfactant was cleaved with 0.5% trifluoroacetic acid (TFA) for 1 h at RT. Digests were centrifuged at 18,000×*g* for 15 min and the supernatant dried. Dried peptides were resuspended in 1 ml 5% formic acid per quantitation channel. Peptides were stable isotope dimethyl labelled according to Boersema et al.^52^. Briefly, labelling was carried out in 200 mg C_18_ columns. Columns were first conditioned with acetonitrile followed by buffer A (0.6% (v:v) acetic acid). Peptides were loaded onto columns and washed with buffer A before being labelled with 5 × 1 ml of either 4% (v:v) CH_2_O, 0.6 M NaBH_3_CN (light), 4% (v:v) CD_2_O, 0.6 M NaBH_3_CN (medium) or 4% (v:v)^13^CD_2_O, 0.6 M NaBD_3_CN (heavy) in 50 mM sodium phosphate pH 7.4. Columns were washed with buffer A and peptides eluted with 50% (v:v) acetonitrile 0.6% (v:v) acetic acid. Samples were mixed according to a 1:1 protein ratio and peptides dried.

### Midgut lysis, digestion and peptide labelling

Midguts were dissected from 10-day-old non-blood-fed female *Ae*. *aegypti* and immediately frozen at −80 °C. For each line (uninfected or *w*Mel infected), midguts from 20 mosquitoes were used for a biological replicate. Midguts were lysed by sonicating 3 × 15 s in 8 M urea and 50 mM TEAB. Protein was measured by BCA assay (Pierce). Samples were reduced with 5 mM DTT for 30 min at 50 °C and alkylated with 15 mM Iodoacetamide for 30 min in the dark. Urea was diluted to 1.5 M with 50 mM TEAB and trypsin/Lys-C (Promega) added at an enzyme/protein ratio of 1:25. Proteins were digested overnight at 37 °C. Digests were acidified with addition of TFA to 0.5% (v:v) and clarified by centrifugation for 7 min at 18,000×*g*. Peptides were desalted using Strata 50 mg C_18_ cartridges (Phenomenex). Cartridges were prepared by passing through 3 ml acetonitrile, 2 ml aqueous 80% (v:v) acetonitrile 0.1% (v:v) TFA and 2 ml aqueous 0.1% (v:v) TFA. Peptides were loaded and cartridges washed with 2 × 0.25 ml aqueous 0.1% TFA. Peptides were eluted with aqueous 80% (v:v) acetonitrile 0.1% (v:v) TFA and dried. Peptides were resuspended in 50 mM TEAB at a concentration of 43 µg/100 µl. A 100 µl aliquot of peptide solution from each sample was labelled using TMT reagents (TMT-10plex, Thermo Fisher). For each sample, 0.8 mg of TMT reagent was resuspended in 41 µl acetonitrile and added to peptides, and labelling took place over 1 h at RT. Excess labelling reagent was quenched with 8 µl aqueous 5% (v:v) hydroxylamine for 15 min at RT. Labelled peptides were dried prior to high pH reversed-phase fractionation.

### Peptide fractionation

Labelled peptides from cell line digests were fractionated by OFFGEL peptide fractionation, carried out according to the manufacturer’s instructions. Briefly, peptides were resuspended in 720 µl water per replicate and mixed with OFFGEL solution containing pH 3-11 ampholytes. Then, 3 mg of peptide was fractionated into 24 wells over a 24 cm pH 3-11 immobilised pH gradient strip. Fractions were acidified with 0.1% formic acid and desalted with StageTips.

Labelled peptides from midgut digests were fractionated using high pH reversed-phase spin columns according to the manufacturer’s instructions (Pierce). Column was washed twice with 300 µl acetonitrile and twice with aqueous 0.1% (v:v) TFA. Peptides were resuspended in 300 µl aqueous 0.1% (v:v) TFA and applied to the column by centrifugation for 2 min at 3,000×*g*. Peptides were washed with 300 µl water, then 300 µl 5% acetonitrile 0.1% triethylamine and eluted with 300 µl each of aqueous (v:v) 10, 12.5, 15, 17.5, 20, 22.5, 25, 50% acetonitrile 0.1% triethylamine. Fractions were dried down for analysis by mass spectrometry.

### Mass spectrometry

Peptides from cell line samples were resuspended in 0.1% formic acid and resolved on a C_18_ reverse-phase PepMap RSLC 50 cm × 75 µm×2 µm, 100 Å Easy-Column (Thermo Scientific) using a linear gradient of 5–55% Buffer B (aqueous 80% (v:v) acetonitrile, 0.1% (v:v) formic acid) at 300 nl/min over 130 min run on an Ultimate 3000 RSLC nano (Dionex) system coupled to QExactive mass spectrometer (Thermo Scientific). The mass spectrometer was operated in a ‘Top 10’ data-dependent acquisition mode with dynamic exclusion enabled (40 s). Survey scans (mass range *m/z* 300–1,650) were acquired at a resolution of 70,000 at 200 Th with the 10 most abundant multiply charged (+2 and +3) ions selected with a 3 Th isolation window for high-energy collisional dissociation (HCD) fragmentation. Tandem mass spectrometry (MS/MS) scans were acquired at a resolution of 17,500 at 200 Th.

Peptides from midgut samples were resuspended in 0.1% formic acid and loaded onto an UltiMate 3000 RSLCnano HPLC system (Thermo) equipped with a PepMap 100 Å C_18_, 5 µm trap column (300 µm × 5 mm, Thermo) and a PepMap, 2 µm, 100 Å, C_18_ Easy Nanonanocapillary column (75 μm × 150 mm, Thermo). The trap wash solvent was 0.05% (v:v) aqueous TFA and the trapping flow rate was 15 µl/min. The trap was washed for 3 min before switching flow to the capillary column. Separation used gradient elution of two solvents: solvent A, aqueous 1% (v:v) formic acid; solvent B, aqueous 80% (v:v) acetonitrile containing 1% (v:v) formic acid. The flow rate for the capillary column was 300 nl/min and the column temperature was 40 °C. The linear multi-step gradient profile was: 3–10% B over 8 min, 10–35% B over 125 min, 35–65% B over 50 min, 65–99% B over 7 min and then proceeded to wash with 99% solvent B for 4 min. The column was returned to initial conditions and re-equilibrated for 15 min before subsequent injections.

The nanoLC system was interfaced with an Orbitrap Fusion hybrid mass spectrometer (Thermo) with an EasyNano ionisation source (Thermo). Positive electrospray ionisation (ESI)-MS, MS^2^ and MS^3^ spectra were acquired using Xcalibur software (version 4.0, Thermo). Instrument source settings were: ion spray voltage, 1,900 V; sweep gas, 0 Arb; ion transfer tube temperature, 275 °C. MS^1^ spectra were acquired in the Orbitrap with: 120,000 resolution, scan range: *m/z* 380–1,500; automatic gain control (AGC) target, 2e^5^; max fill time, 50 ms. Data-dependant acquisition was performed in top speed mode using a 4 s cycle, selecting the most intense precursors with charge states >1. Dynamic exclusion was performed for 50 s post-precursor selection and a minimum threshold for fragmentation was set at 3e^4^. MS^2^ spectra were acquired in the linear ion trap with: scan rate, turbo; quadrupole isolation, 1.2 *m/z*; activation type, collision-induced dissociation; activation energy: 35%; AGC target, 1e^4^; first mass, 120 *m/z*; max fill time, 50 ms. MS^3^ spectra were acquired in multi notch synchronous precursor mode (SPS^3^), selecting the 5 most intense MS^2^ fragment ions between 400 and 1,000 *m/z*. SPS^3^ spectra were measured in the Orbitrap mass analyser using: 50,000 resolution, quadrupole isolation, 2 *m/z*; activation type, HCD; collision energy, 65%; scan range: *m/z* 110–500; AGC target, 5e^4^; max fill time, 86 ms. Acquisitions were arranged by Xcalibur to inject ions for all available parallelisable time.

### MS data analysis

MS data files from cell line samples were de-isotoped and charge deconvoluted with Proteome Discoverer 1.4 (Thermo). Files were searched using Mascot 2.4.1 against a concatenated and reversed decoy *Ae*. *aegypti* and *Wolbachia w*Mel database containing 18,082 sequences. Cysteine carbamidomethylation and protein N-terminal acetylation were set as fixed modifications. Oxidation (M), dimethyl (K, N-termini), dimethyl-4 (K, N-termini) and dimethyl-8 (K, N-termini) were set as variable modifications. Precursor mass tolerance was 20 p.p.m., and fragment mass tolerance was 0.1 Da. Two mis-cleavages were allowed. Peptide cutoff score was 10 and protein relevance threshold was 20, false discovery rate (FDR) was 1.23%. The structure of Proteome Discoverer.msf files was viewed with DbVisualizer (DbVis Software AB). The ‘PrecursorIonQuanResults’ table was exported. For each peptide with a quantitation status of ‘used’, the ‘QuanResultID’ was used to obtain light, medium and heavy peak areas, summed within each ‘QuanChannelID’. For each biological replicate, proteins with >=3 ‘QuanResultID’ associated were retained. Peptide intensities for each protein were summed within quantitation channels. Differentially expressed proteins were determined using LIMMA^51^, with normalizeWithinArrays (method=loess) and normalizeBetweenArrays (method=quantile). *P-*values were calculated for protein fold changes, adjusted for multiple hypothesis testing with the Benjamini–Hochberg method^53^, and fold changes with an adjusted *P-*value < 0.05 were accepted. From the cell line, 3,785 proteins, representing ~28% of the proteome, were identified and quantified in at least 3 biological replicates.

For midgut-derived TMT data peak lists were converted from centroided .raw to .mgf format using Mascot Distiller (version 2.6.1, Matrix Science) and MS^3^ spectra were concatenated into their parent MS^2^ spectra for database searching. Mascot Daemon (version 2.5.1, Matrix Science) was used to combine .mgf files and search against a subset of the UniProt database containing *Ae*. *aegypti* and *Wolbachia* w Mel proteins (17,811 sequences) using a locally running copy of the Mascot program (Matrix Science Ltd, version 2.5.1). Search criteria specified: Enzyme, trypsin; Fixed modifications, Carbamidomethyl (C), TMT6plex (N-term, K); Variable modifications, Oxidation (M); Peptide tolerance, 5 p.p.m.; MS/MS tolerance, 0.5 Da; Instrument, ESI-TRAP. The Mascot .dat result file was imported into Scaffold Q + (version 4.7.5, Proteome Software) and a second search run against the same database using X!Tandem was run. Protein identifications were filtered to require a maximum protein and peptide FDR of 1% with a minimum of two unique peptide identifications per protein. Protein probabilities were assigned by the Protein Prophet algorithm^54^. Proteins that contained similar peptides and could not be differentiated based on MS/MS analysis alone were grouped to satisfy the principles of parsimony. Proteins sharing significant peptide evidence were grouped into clusters. Quantification of relative protein abundance was calculated from TMT reporter ion intensities using Scaffold Q +. TMT isotope correction factors were taken from the document supplied with the reagents by the manufacturer. Normalisation was performed iteratively (across samples and spectra) on intensities, as described in ref. ^55^. Medians were used for averaging. Spectra data were log-transformed, pruned of those matched to multiple proteins and weighted by an adaptive intensity weighting algorithm. Differentially expressed proteins were determined by applying permutation test with unadjusted significance level *p* < 0.05 corrected by Benjamini–Hochberg for FDR estimation. The mass spectrometry proteomics data have been deposited to the ProteomeXchange Consortium^56^ via the PRIDE partner repository with the data set identifier PXD003429 (Aag2 cell data) or PXD006239 (midgut data).

### Cholesterol quantification

Aag2 cells stably infected with *w*MelPop and a rifampicin cured negative line were grown to confluency in six-well plates. Cells were washed twice with PBS prior to harvesting by scraping. In all, 12 wells of each cell type were harvested for cholesterol measurements. Then, 200 µl of hexane/isopropanol 3:2 was added to the cell pellet from half a well to extract cholesterol. Extracts were centrifuged at 10,000×*g* for 5 min at RT, the supernatant was dried in a vacuum centrifuge overnight and extracts resuspended in 100 µl reaction buffer (0.1 M potassium phosphate pH 7.4, 0.05 M NaCl, 5 mM cholic acid, 0.1% Triton X-100, Thermo Fisher). Free cholesterol was quantified using the Amplex Red Cholesterol Assay Kit (Thermo Fisher), and esterified cholesterol was quantified by including cholesterol esterase in the reactions. Cholesterol quantities were normalised to total input protein. Input protein was measured by resuspending the protein pellet left after hexane/isopropanol cholesterol extraction in 100 µl RIPA buffer (Sigma). Protein content was measured using the BCA assay (Thermo Scientific).

### Imaging

Uninfected and infected *w*MelPop Aag2 cells were seeded at 3 × 10^5^ cells/well in 1 ml FBS and penicillin/streptomycin supplemented Schneider’s *Drosophila* medium in a 24-well poly-l-lysine-coated plate and allowed to adhere overnight. Cholesterol was labelled according to Sankaranarayanan et al.^57^; briefly, labelling media was prepared by adding 3.47 ml of 20 mM methyl-beta-cyclodextrin (Sigma) in unsupplemented Schneider’s *Drosophila* medium to 1 mg of 23-(dipyrrometheneborondifluoride)-24-norcholesterol (TopFluor cholesterol, Avanti Polar Lipids) to achieve a 1:40 cholesterol/cyclodextrin molar ratio. This mixture was sonicated for 6 × 5 s pulses. The sample was then centrifuged at 19,000×*g* for 10 min to remove undissolved cholesterol. TopFluor cholesterol was then diluted 1:1 in unsupplemented Schneider’s *Drosophila* medium. This was then added to the cells and incubated for 30 min to allow labelling to occur. Labelling media were then removed and either PBS or 0.1 mM of 2HPCD was added. After 48 h, the cells were washed with PBS before Vecta shield mounting media with 4',6-diamidino-2-phenylindole (DAPI; Vectorlabs) was added. Images were then acquired using a Zeiss LSM 880 confocal microscope (Zeiss) with a 63× objective. Cholesterol was imaged using a 488 nm laser, with GaAsP detectors. Nuclei stained with DAPI were imaged using a 405 nm laser with GaAsP detector. All settings were obtained by first imaging uninfected Aag2 cells incubated in PBS as a standard control ensuring all data were correlated. *Z*-stack images were collected and analysed using Zen2 software (Zeiss) to create a 3D reconstruction of imaged cells after de-convolution was carried out.

Quantification was carried out by imaging 5 independent ×64 images from 5 independent wells on a 24-well optical plate. Images were analysed using Cell Profiler. A global image threshold was set using the Otsu method and images were analysed in order to identify the number of nuclei and the number of green spots corresponding to TopFluor staining. Data are presented as the number of spots per cell.

For Rab7 staining, Aag2 *w*MelPop cells were fixed with 2.5% paraformaldehyde and permeabilised in PBS containing 0.01% Triton-X for 15 min. Cells were blocked for 1 h with 5% serum before incubation with Rab7 antibody (Abcam, ab50533, 1:500). Images were obtained as stated above.

For Nile Red staining, cells were fixed and permeabilised as above. Cells were then incubated for 10 min in 10 µg/ml of Nile Red. Nile Red is known to fluoresce in green/yellow where lipid droplets are present^39^. Images were then acquired using a Zeiss LSM 880 confocal microscope (Zeiss) with a 63× objective as above with the 514 laser. Images were processed in Zen blue and lipid droplets measured.

### DENV-2 propagation, infection and quantification

DENV-2 New Guinea C strain (Public Health England, 0006041v), propagated in Vero cells, was diluted 1:100, one round of propagation was carried out in C6/36 cells (ECACC catalogue no. 89051705) for 5 days and the resulting virus stock was centrifuged for 7 min at 400×*g* and quantified by fluorescent focus assay (FFA)^58^. For the FFA, Vero cells were plated at a density of 0.9 × 10^5^ cells/well in 96-well special optics plates (Corning). The following day, 10-fold serial dilutions of DENV containing supernatant were prepared using DMEM with 2% FBS. Medium was removed from Vero cells and 50 µl of each serial dilution was added to wells. Plate was placed on an orbital shaker at 20 r.p.m. and left at 37 °C for DENV to bind for 45 min. DENV was removed and 150 µl of overlay medium/well (DMEM with 0.8% carboxymethylcellulose, 5% FBS, 2 mM l-glutamine, 200 units/ml penicillin 200 µg/ml streptomycin) was added. Plates were left for 48 h, overlay medium was removed and cells washed twice in PBS. Cells were fixed with methanol for 10 min at 4 °C, washed with 150 µl PBS and permeabilised for 5 min with 150 µl 0.1% Triton X-100 (Fisher). Permeabilisation solution was removed and 35 µl of 1:500 anti-DENV antibody was added (MAB8705, Millipore), diluted in 0.2% bovine serum albumin (BSA) PBS. Plates were incubated for 1.5 h at RT, followed by 3 washes of 100 µl with 0.2% BSA PBS. Cells were incubated with 35 µl of 1:400 secondary antibody (Goat anti-mouse Alexa Fluor 488, A-11001, Thermo Fisher) in 0.2% BSA PBS for 1 h at RT. Cells were washed 3 times with 0.2% BSA PBS and fluorescent foci were counted using a fluorescent microscope or plates were imaged with a Typhoon FLA 9500 scanner using filter settings for Cy2. Plate images were processed in ImageJ, the ‘Analyze Particles’ feature was used to count fluorescent foci.

For the DENV rescue experiments, Aag2 cells were seeded in 96-well tissue culture plates (Corning) at a density of 150,000 cells/ml. The next day treatment commenced with 2-hydroxypropyl-β-cyclodextrin (Sigma) at the indicated concentrations in cell culture medium. After 48 h of treatment, cell culture medium was removed, cells were washed with 100 µl PBS and DENV was added in Schneider’s *Drosophila* medium without FBS at a multiplicity of infection of 0.1 in a volume of 35 µl/well. Plates were placed on an orbital shaker set at 20 r.p.m. for 2 h at RT. Virus was removed and normal Schneider’s *Drosophila* medium was added with 10% FBS and 200 units/ml penicillin 200 μg/ml streptomycin (Sigma). Plates were incubated at 28 °C for 5 days after which cells were harvested in 100 µl Tri reagent (Sigma) and RNA extracted as described below. For quantification of DENV by FFA, supernatant was harvested 7 days post infection.

### Mosquito line and rearing

A *w*Mel-infected *Ae*. *aegypti* line was created by microinjecting wild-type embryos from a Malaysian-derived line with cytoplasm from an Indonesian-derived (UJU) *w*Mel *Ae*. *albopictus *line^6^. The line was outcrossed with males of the wild-type line for three generations, and maintained for ∼20 generations. *Wolbachia* density in the *w*Mel line midguts was 7.8 + –4.6 (S.D., *n* = 5) Wolbachia surface protein/homothorax (*wsp/HTH*) as measured by qPCR (see below). Maternal transmission was 100% as determined by PCR. *Ae*. *aegypti* wild type and *w*Mel-infected lines were maintained at 27 °C at 70% humidity.

### RNA/DNA extraction and qPCR

For estimation of *Wolbachia* density, DNA was extracted using phenol/chloroform from three independent samples of the Aag2 *w*MelPop cell line. Absolute *Wolbachia* density was measured by qPCR using primers targeting *wsp* (forward: 5′-GCATCTTTTATAGCTGGTGG-3′, reverse: 5′-AAAGTCCTTCAACATCAACCC-3′, normalised to host *HTH* (forward: 5′-TGGTCCTATATTGGCGGAGCTA-3′, reverse: 5′-TCGTTTTTGCAAGAAGGTCA-3′)^16^. Relative *Wolbachia* density in the 2HPCD experiments was measured by qPCR using primers targeting *wsp*, normalised to host rp49 (forward: 5′-GCTATGACAAGCTTGCCCCCA-3′, reverse: 5′-TCATCAGCACCTCCAGCTC-3′)^59^.

### Data availability

The data from mass spectrometry are deposited at the ProteomeXchange Consortium via the PRIDE partner repository with the data set identifiers PXD003429 and PXD006239. The authors declare that all other data supporting the findings of this study are available within the article and its Supplementary Information files, or are available from the authors on request.

## Electronic supplementary material

Supplementary Information

Supplementary Dataset 1

Supplementary Dataset 2

Supplementary Dataset 3
