# Supplementary material for: Perturbed cholesterol and vesicular trafficking associated with dengue blocking in Wolbachia-infected Aedes aegypti cells
Source: Nat Commun. 2017 Sep 13;8:526. doi: 10.1038/s41467-017-00610-8 (PMC5597582; doi:10.1038/s41467-017-00610-8)
Supplement: Supplementary file 1 — Supplementary Information [file 41467_2017_610_MOESM1_ESM.pdf]

File name: Supplementary Information
Description: Supplementary Figures, Supplementary Tables and Supplementary References

File name: Supplementary Data 1
Description: All identified and quantified proteins, Aag2 cells.

File name: Supplementary Data 2
Description: All differentially expressed proteins with functional annotation.

File name: Supplementary Data 3
Description: All identified and quantified proteins from *Ae. aegypti* midguts.

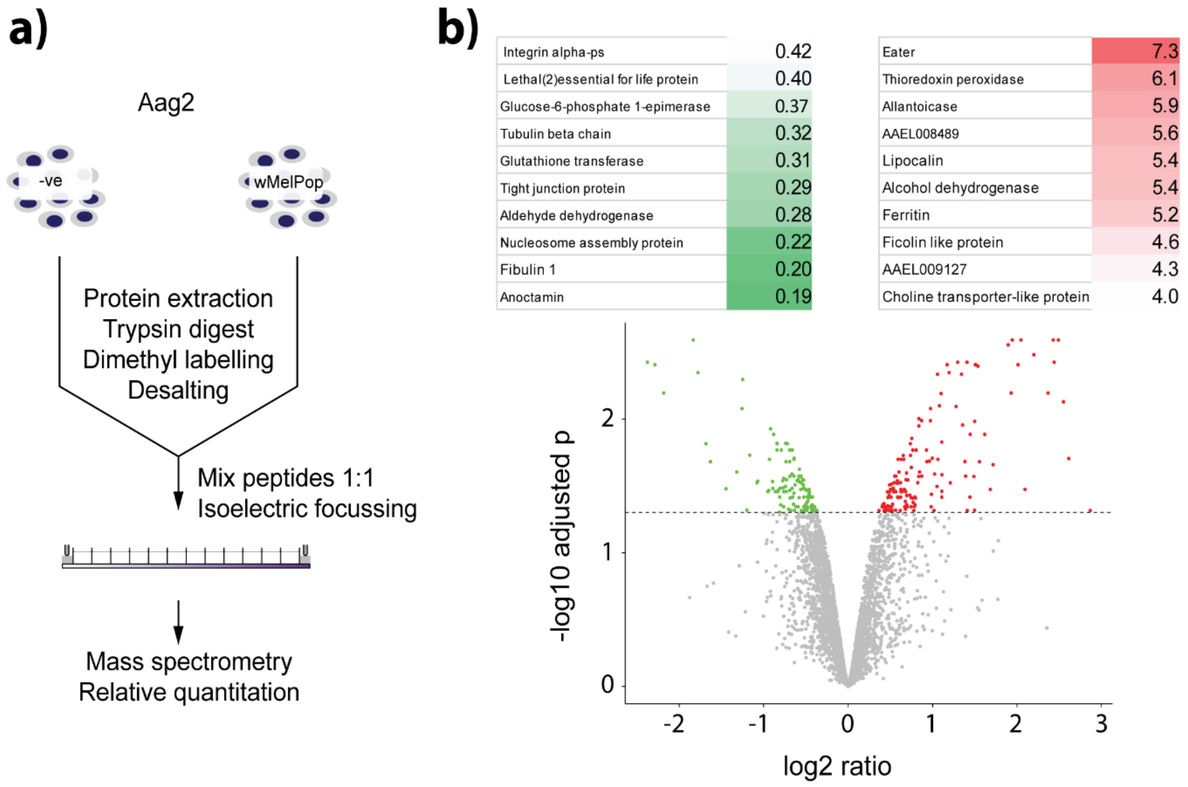

Supplementary Figure 1. Proteomic characterisation of the host response to *Wolbachia* in *Aedes aegypti* (Aag2) cells. A) Experimental workflow. Aag2 cells (uninfected or containing *w*MelPop) were lysed in acid labile surfactant, protein was extracted and digested with trypsin. Peptides were dimethyl labelled, mixed in a 1:1 protein ratio and fractionated by OFFGEL isoelectric focussing prior to identification and relative quantification by mass spectrometry. This workflow was applied to 4 independent biological replicates. B) Differentially regulated proteins in *w*MelPop infected cells. Most up-(red) or down-(green) regulated proteins (top). Volcano plot of quantified proteins. The ratio of peptide intensities for each protein in infected cells vs. uninfected cells is plotted against the adjusted p value calculated from differential expression analysis (LIMMA).

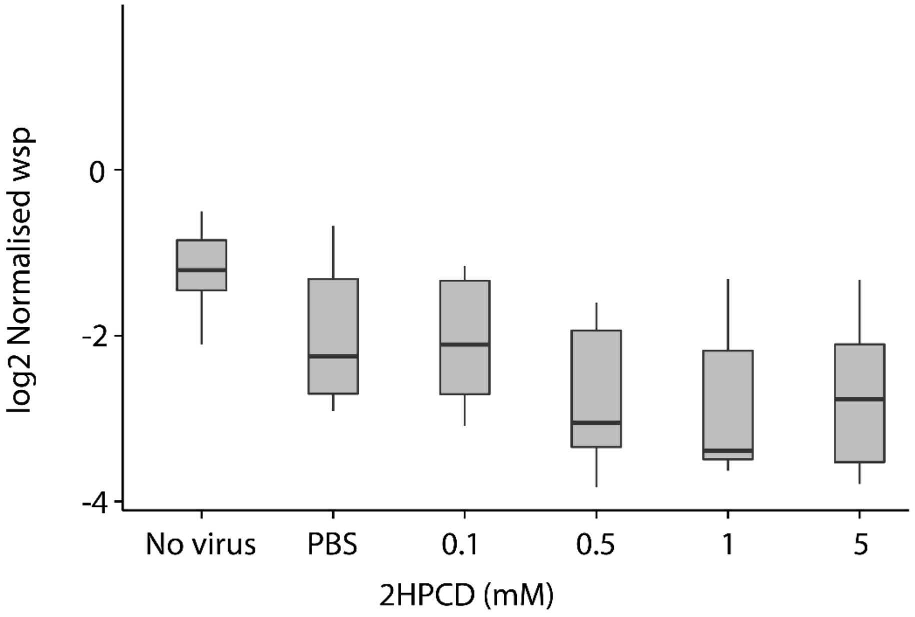

Supplementary Figure 2. Relative quantification of the *Wolbachia* gene *wsp*, normalised to host *rp49*, by qPCR in the 2HPCD treated cells. No significant change in normalised *wsp* levels was found as determined by ANOVA corrected by Tukey HSD test, *n*=6.

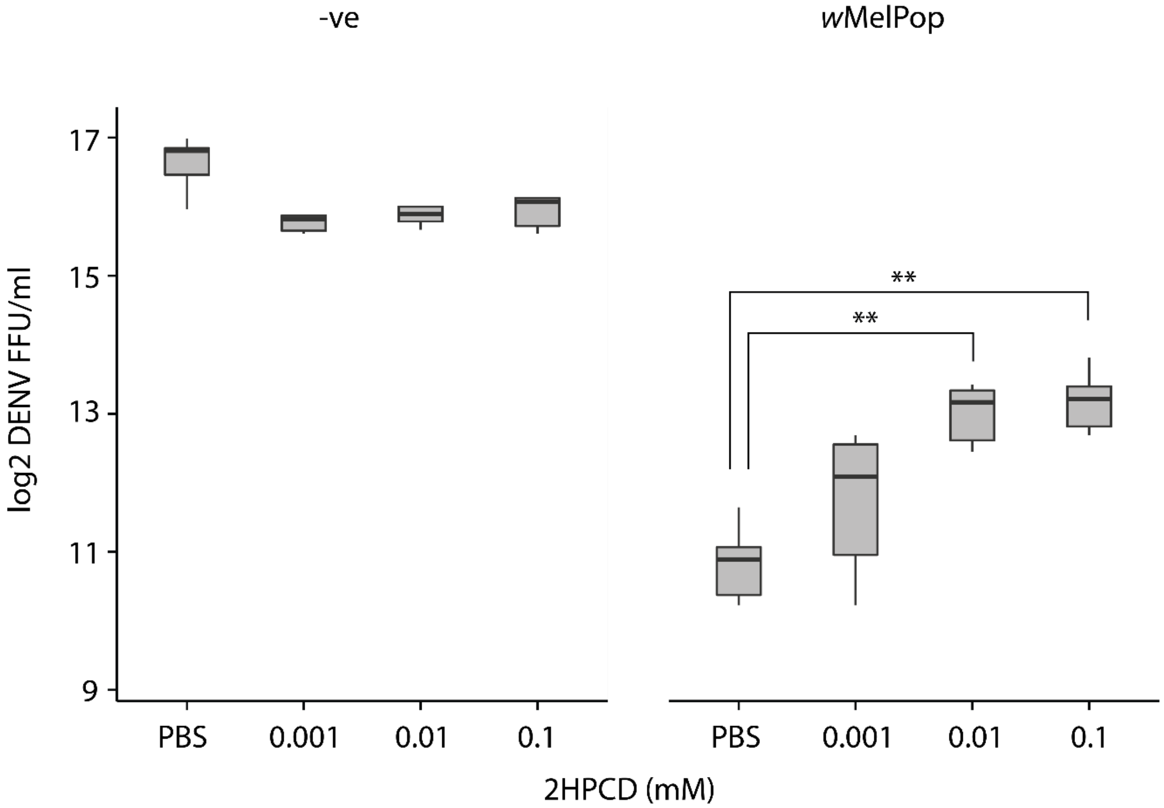

Supplementary Figure3. Pre-treatment of Aag2 cells with 2-hydroxypropyl beta cyclodextrin (2HPCD) reverses *Wolbachia* induced inhibition of DENV replication. Uninfected (-ve) or *w*MelPop infected cells were pre-treated for 48hrs with various concentrations of 2HPCD before being infected with DENV at a multiplicity of infection of 0.1. Supernatant was harvested 7 days post-infection and infectious DENV particles were quantified by Fluorescent Focus Assay. The statistically significant differences are indicated, calculated using ANOVA corrected by Tukey HSD, *n=6*.

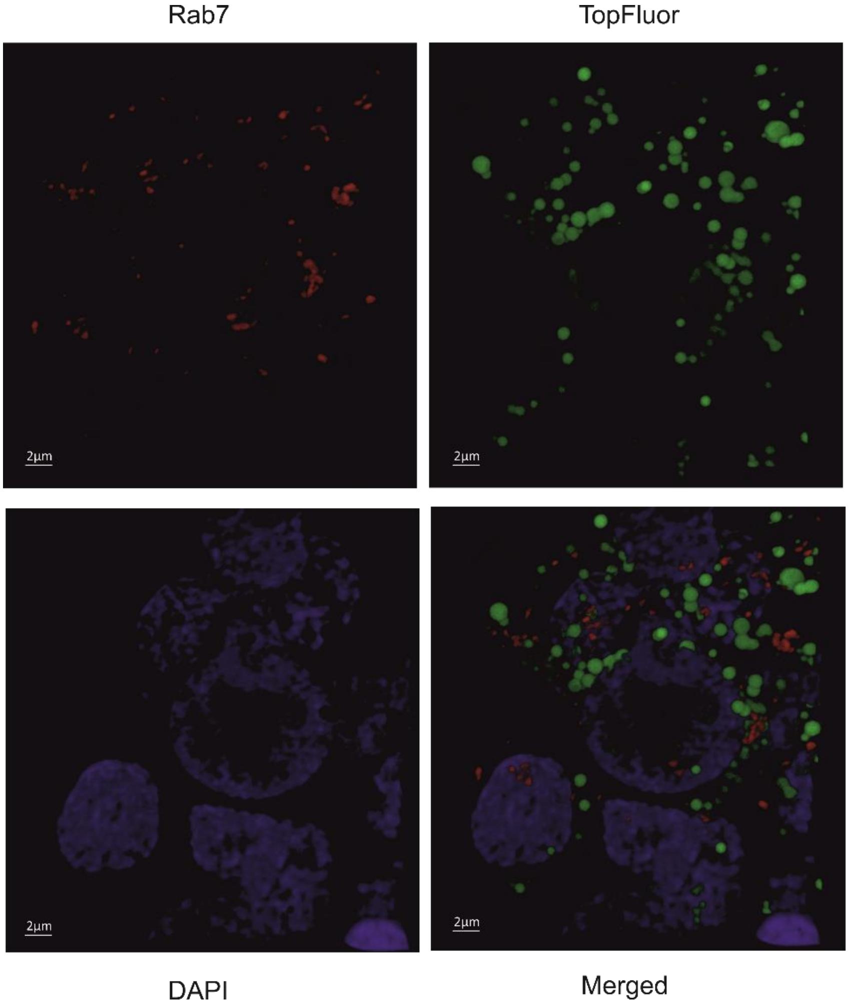

Supplementary Figure4. Imaging the late endosome marker Rab7 and TopFluor cholesterol in Aag2 *w*MelPop cells. Scale bar indicates 2 µm.

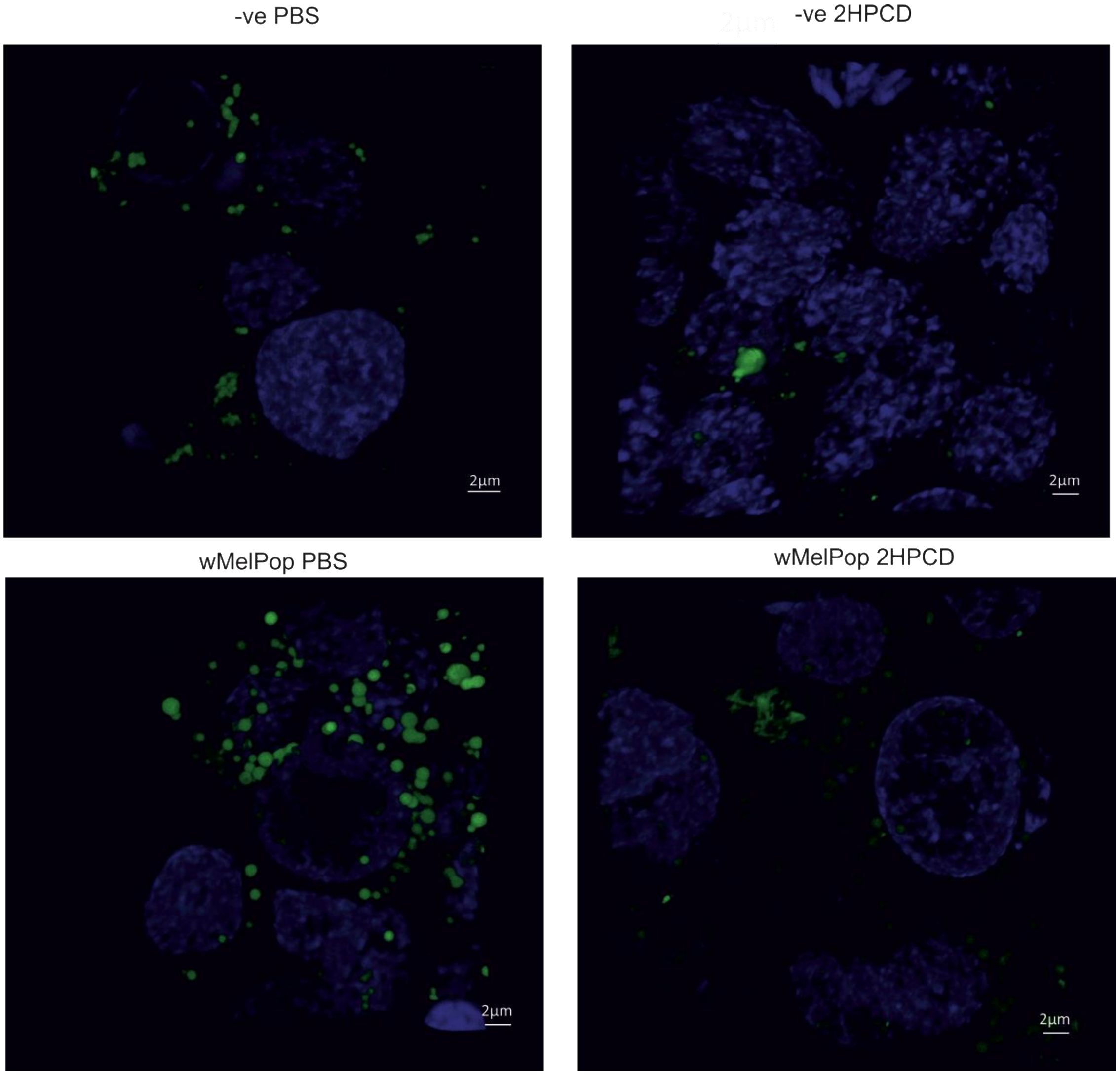

Supplementary Figure5. Cholesterol turnover imaged using TopFluor cholesterol. Uninfected and *w*MelPop infected cells were pre-treated with either PBS or 0.1mM 2HPCD for 24 hrs. Cells were then labelled with TopFluor cholesterol for 30 mins and subsequently incubated with either PBS or 0.1mM 2HPCD for 24hrs before imaging by fluorescence confocal microscopy. Z-stack images were taken and 3D reconstructions made in order to look at overall accumulation and/or diffusion of TopFluor in cells upon treatment. Scale bar indicates 2 μm.

Replicate	Protein groups	Unique peptides
1	4179	36,231
2	3940	52,072
3	4290	39,693
4	4281	41,061

Supplementary Table 1. Summary of quantified proteins and identified peptides in each biological replicate. Proteins were required to have at least 3 associated quantitation events per biological replicate and be identified in at least 3 biological replicates. The *Aedes aegypti* genome contains approximately 12,549-13,594 genes as detected by RNA sequencing experiments[1]. In this proteomic screen, 3785 proteins, representing ~28% of the proteome, were identified and quantified in at least 3 biological replicates.

Fold change *w*MelPop/-ve

Autophagy	
Vacuolar ATP synthase subunit c	0.75
Putative cysteine protease required for autophagy (ATG4B)	0.67
Tuberous sclerosis complex 2 (TSC2)	0.45

ER/unfolded protein response	
UDP-glycosyltransferase family	2.12
UDP-glycosyltransferase family	1.98
Subunit of the N-oligosaccharyl transferase complex	1.78
Thioredoxin/protein disulfide isomerase	1.75
Poly polymerase 16	1.72
Putative udp-glucuronosyl and udp-glucosyl transferase	1.63
Oligosaccharyl transferase	1.51
Oligosaccharyl transferase	1.42
Truncated ER mannose-binding lectin	1.41
Putative translocon-associated complex trap gamma subunit	1.37
Putative calnexin 99a	1.35
Anoctamin	0.19

Iron homeostasis	
Ferritin (AAEL004335)	5.16
Ferritin (AAEL007383)	1.99
Transferrin domain	1.73

Lipid metabolism	
Apolipoprotein D	5.42
Niemann-Pick type C-2e (NPC2)/ML33	2.90
Putative lipid exporter abca1 (AAEL012701)	2.84
Putative lipid exporter abca1 (AAEL015146)	2.65
Saposin	1.79
Putative peroxisomal phytanoyl-coa hydroxylase	1.74
Putative fatty acid desaturase	1.67
Putative microsomal triglyceride transfer protein	1.62
Putative mitochondrial fatty acid anion carrier	1.45
Putative lipid particle	1.39
Fatty acid synthase	0.75
Protein phosphatase 5	0.74
Acetyl-coa carboxylase	0.69
Allergen, putative	0.60
Low-density lipoprotein receptor (ldl)	0.42

Redox homeostasis	
Thioredoxin peroxidase	6.11
Putative purple acid phosphatase	2.66
Protein disulfide isomerase	1.33
Putative tlpa-like family, thioredoxin like protein	0.69

Vesicular trafficking	
Putative lysosomal protein ncu-g1	2.72
Putative snare protein pep12/vam3/syntaxin 7/syntaxin 17	2.43
Wurst	1.83
Mucolipin-3	1.62
Vacuolar sorting protein	1.54
Putative rush hour	1.40
Syntenin	1.35
Rabkinesin-6	0.74
Tomosyn	0.70
n-myc downstream regulated (ndrg1)	0.67
myosin V	0.54

Wolbachia recognition/immune response	
Eater	7.30
Ficolin like protein	4.59
Homologous to osiris2	3.81
Serine protease inhibitor like	2.85
Scramblase 1, isoform C	2.62
Putative peptidylglycine alpha-amidating monooxygenase	2.03
Scavengor receptor	2.02
Galectin	1.66
Serine palmitoyltransferase	1.59
leucine-rich immune protein (TM)	0.60

Supplementary Table 2. Categorised differentially expressed proteins in *w*MelPop infected Aag2 cells vs. uninfected cells. Differential expression analysis was performed using LIMMA with an adjusted p value cut-off of 0.05. Homologues for unannotated proteins were obtained with BLASTP searches.

Fold change *w*Mel/-ve

Autophagy	
Putative puromycin sensitive aminopeptidease	1.22
Rabconnectin	1.06
Vacuolar H+-ATPase V1 sector subunit D	0.92
Cathepsin L	0.85

ER/unfolded protein response	
Vacuolar ATPase assembly integral membrane protein	1.36
Alpha-glucosidase	1.26
Dolichyl-diphosphooligosaccharide--protein glycosyltransferase subunit stt3b	1.21
Dolichyl-diphosphooligosaccharide--protein glycosyltransferase subunit DAD1	1.21
tRNA-splicing ligase RtcB	1.21
Putative mannose lectin ergic-53	1.2
Putative calnexin 99a	1.16
Putative yip1	1.16
Reticulon-like protein	1.16
Putative proteasome non-atpase 26s subunit	1.13
Putative sec23-binding domain of sec16	1.12
Heat shock protein	1.11
Peptidylprolyl isomerase	1.09
Putative transport and golgi organization protein 1	1.09
Gp210	0.91

Iron homeostasis	
Ferritin	1.28
Ferritin	0.85

Lipid metabolism	
Niemann-Pick type C2 (AAEL015136)	2.3
Niemann-Pick type C2 (AAEL009760)	2.06
Sterol carrier protein 2-like 2	1.85
Niemann-pick C1	1.35
Palmitoyltransferase	1.33
Lipid droplet associated hydrolase	1.33
Sterol carrier protein 2-like 3 variant 1	1.27
Putative oxysterol binding protein	1.24
Phosphoacetylglucosamine mutase	1.21
Putative very long-chain acyl-coa synthetase/fatty acid transporter	1.17
Glycerol-3-phosphate acyltransferase 3-like	1.15
Putative 85 kDa calcium-independent phospholipase a2	1.13
Putative lipid particle	0.89
Sterol carrier protein-X	0.88
Acyl-coenzyme A oxidase	0.88
Putative fatty acid desaturase	0.82

Redox homeostasis	
Constitutive coactivator of peroxisome proliferator-activated receptor gamma	1.42
Regucalcin	1.26
Superoxide dismutase	0.91

Vesicular trafficking	
Putative syntaxin 1a	1.29
Putative golgi protein	1.24
Putative neurobeachin	1.15
Vesicle protein sorting-associated	1.15
Use1	1.13
Putative vesicle coat complex copi alpha subunit	1.07
Putative nipsnap	0.81

Lysosomal storage disorder related	
Sphingomyelin phosphodiesterase	1.88
Ceramidase	1.54
Alpha-mannosidase	1.38
Putative palmitoyl protein thioesterase	1.21
Plasma alpha L fucosidase	1.18
Sphingomyelin phosphodiesterase 2	1.1

Wolbachia recognition/immune response	
Defensin-A	2.95
Phosphatidylethanolamine binding protein	1.75
Putative apolipophorin III	1.29
Apolipophorin	1.19

Supplementary Table 3. Categorised differentially expressed proteins in *w*Mel infected vs wt *Aedes aegypti* midguts. Differential expression analysis was performed with Scaffold, significance threshold for fold changes was $p < 0.0005$ (permutation test).

Supplementary References

1. Juneja, P. et al. Exome and transcriptome sequencing of Aedes aegypti identifies a locus that confers resistance to Brugia malayi and alters the immune response. *PLoS Pathog* **11**, e1004765 (2015).
